# Effects of Different Yeast Strains on Fermentation Characteristics, Volatile Flavor Compounds, and Sensory Quality of Xinjiang *Ziziphus jujuba* ‘Huizao’ Wine

**DOI:** 10.3390/microorganisms14061178

**Published:** 2026-05-23

**Authors:** Bei Zhao, Liubin Huang, Qi Zuo, Yanxia Fan, Muhammad Yousuf Adnan, Sen Wang, Fengxia Shao

**Affiliations:** 1 College of Forestry, Central South University of Forestry and Technology, Changsha 410004, China; 20231100016@csuft.edu.cn (B.Z.); 20230100008@csuft.edu.cn (L.H.); 20231200165@csuft.edu.cn (Q.Z.); t20192461@csuft.edu.cn (Y.F.); myadnan@uok.edu.pk (M.Y.A.); 2 The Belt and Road International Union Research Center for Tropical Arid Non-Wood Forest in Hunan Province, Changsha 410004, China; 3 Yuelushan Laboratory, Changsha 410128, China; 4 Department of Botany, University of Karachi, Karachi 75270, Pakistan

**Keywords:** Xinjiang *Ziziphus jujuba* ‘Huizao’ wine, indigenous preserved yeast, volatile flavor compounds, sensory quality, *Saccharomyces cerevisiae*, non-*Saccharomyces* yeast

## Abstract

Xinjiang *Ziziphus jujuba* ‘Huizao’ wine, a characteristic fruit wine in China, is facing industrial bottlenecks such as flavor homogenization and lack of specialized fermentation yeasts, which limits its high-quality development. To solve these problems, four laboratory-preserved indigenous yeast strains (NZ5, NZ6, BH4, BH2) were compared with four commercial strains (FR, RW, RA, SY) in terms of fermentation dynamics, volatile flavor compound synthesis (HS-SPME-GC-MS/GC-FID), and sensory quality to screen the optimal yeast for *Ziziphus jujuba* ‘Huizao’ wine fermentation. Molecular identification revealed that NZ5 and NZ6 belong to *Saccharomyces cerevisiae*, while BH4 and BH2 are closely related to *Pichia kudriavzevii*, respectively, indicating their non-*Saccharomyces* characteristics with distinct metabolic potentials. The results showed that indigenous strains exhibited significantly superior performance to commercial strains: (1) *Saccharomyces* strains NZ5 and NZ6 had higher fermentation efficiency, with 12.5–25% shorter fermentation cycles and 14% higher cumulative CO_2_ release than commercial strains; (2) Non-*Saccharomyces* strain BH4 synthesized the most diverse volatile flavors (99 compounds), with ethyl acetate content reaching 314.92 mg/L, which was 13-fold higher than that of commercial yeast FR (24.09 mg/L). Meanwhile, its phenethyl alcohol content reached 3.12 mg/L, 7.2 times that of commercial yeast RW; (3) Sensory evaluation showed that BH4-fermented wine had the highest score (88.59), significantly higher than commercial strains (63.57–67.67). In conclusion, BH4 is the optimal strain for improving the flavor quality of Xinjiang *Ziziphus jujuba* ‘Huizao’ wine, and NZ5/NZ6 are suitable for efficient industrial fermentation. This study provides valuable microbial resources and technical guidance for the quality improvement and industrial development of Xinjiang characteristic fruit wine.

## 1. Introduction

Jujube (*Ziziphus jujuba* Mill.), a member of the Rhamnaceae family, has been cultivated in China for over 4000 years and is primarily distributed across Xinjiang, Shaanxi, Shanxi, Hebei, and neighboring regions [1,2]. Xinjiang, with its abundant sunlight, large diurnal temperature differences and favorable climatic conditions, has become the core production area for high-quality red dates. As a novel fruit wine product, jujube wine retains the nutritional benefits of jujubes while gaining unique flavors and functional properties through fermentation [3]. It is richer in active components such as cAMP and flavonoids compared to traditional fruit wines [4], offering greater health benefits. However, the jujube wine industry in Xinjiang faces bottlenecks, such as product homogenization and unstable flavor quality, primarily due to the lack of highly efficient, specialized fermentative yeast strains adapted to jujube substrates [5].

Yeast metabolic traits play a decisive role in fermentation efficiency, ethanol production, and aroma compound formation, thereby determining the final sensory quality of fermented beverages [6,7]. However, current domestic and international research on fruit wine yeasts predominantly focuses on traditional fruit wines such as grape wine and cider. The *Saccharomyces cerevisiae* strain EC1118 is widely used in wine production due to its stable fermentation behavior and consistent aroma-forming ability [8]; Feng et al. [9] successfully screened eight yeast strains with low higher alcohols production by treating *Saccharomyces cerevisiae* with atmospheric pressure room-temperature plasma, providing technical support for improving the quality of traditional fruit wines. Although previous studies have made important progress in yeast screening and metabolic regulation, research on strains specifically adapted to the high-sugar, high-acid conditions of Xinjiang jujube wine remains limited. As a result, commercially available yeasts often fail to meet the industry’s requirements for aroma complexity and product stability.

Our research group has previously isolated and preserved a number of indigenous yeast strains from the natural habitat of *Ziziphus jujuba* ‘Huizao’. From this collection, four high-potential yeast strains (NZ6, BH4, NZ5, BH2) were selected based on preliminary evaluations. To systematically assess their suitability for jujube wine production, this study compares these preserved strains with four widely used commercial *Saccharomyces cerevisiae* strains (FR, RW, RA, SY), known for their strong fermentation capabilities, as control strains. The comparison encompasses growth characteristics, stress tolerance, fermentation kinetics, and the synthesis of volatile flavor compounds supplemented by comprehensive sensory evaluation. The objective of the study is to identify yeast strains that combine efficient fermentation performance with superior aroma-forming ability, thereby providing high-quality microbial resources for jujube wine production. The findings aim to offer theoretical guidance and technical support to enhance product quality, reduce flavor inconsistency and promote industrial innovation within the Xinjiang jujube wine industry.

## 2. Materials and Methods

### 2.1. Materials and Reagents

#### 2.1.1. Jujube Samples and Yeast Strains

*Ziziphus jujuba* ‘Huizao’ fruits were harvested from commercial orchards in Aksu Prefecture, Xinjiang Uygur Autonomous Region, China.

Four indigenous yeast strains (NZ5, NZ6, BH4, BH2) were isolated from naturally fermented juice of *Ziziphus jujuba* ‘Huizao’ using the gradient dilution plating method on YPD agar medium supplemented with 0.2 g/L chloramphenicol. All strains were preserved in 30% (*v*/*v*) glycerol at −80 °C in the Key Laboratory of Non-Wood Forestry, Central South University of Forestry and Technology, Changsha, China. The 26S rDNA sequences of these strains were deposited in GenBank with the following accession numbers: NZ5 (PZ386133), NZ6 (PZ386134), BH2 (PZ386132), and BH4 (PZ386131).

The designations of indigenous yeast strains are defined as follows: NZ represents strains isolated from naturally fermented samples of *Ziziphus jujuba* ‘Huizao’, and BH represents strains isolated from the fruit surface of *Ziziphus jujuba* ‘Huizao’. Numbers 5, 6, 2, and 4 are sequential identifiers assigned during strain isolation and preservation.

These four strains were selected from more than 30 yeast isolates based on preliminary evaluation, including growth performance, tolerance to high sugar, high acid, and ethanol, as well as primary fermentation capacity. Only strains with superior fermentation potential were chosen for this study.

Four commercial yeast strains were used as controls: *Saccharomyces cerevisiae* RW and SY were purchased from Angel Yeast Co., Ltd., Yichang, Hubei, (China), and *S. cerevisiae* FR and RA were obtained from Diboshi Co., Ltd., Yantai, Shandong (China).

#### 2.1.2. Reagents and Standards

Food-grade sucrose and citric acid were purchased from Shaanxi Zhenghong Biotechnology Co., Ltd. (Xi’an, China). Analytical-grade sodium hydroxide, hydrochloric acid, sulfuric acid, and anhydrous ethanol were supplied by Sinopharm Chemical Reagent Co., Ltd. (Shanghai, China). All reagents were used without further purification.

#### 2.1.3. Culture Medium

Yeast Extract Peptone Dextrose (YPD) solid medium (g/L) was prepared as follows: yeast extract 10, peptone 20, glucose 20, chloramphenicol 0.2, agar powder 20, with a measured initial pH of 5.8 ± 0.2. YPD liquid medium was prepared with the same composition excluding agar powder. Both media were autoclaved at 121 °C for 20 min and cooled to room temperature before use [10].

### 2.2. Fermentation

#### 2.2.1. Fermentation Process

**Figure 1:**
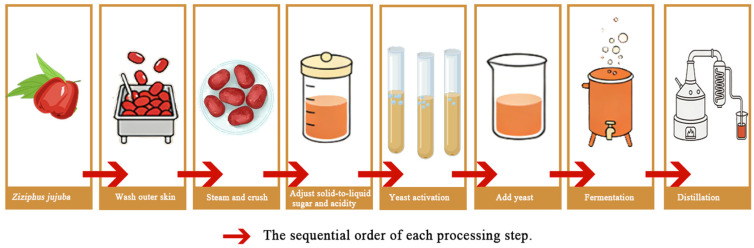
Fermentation Flow Chart of *Z. jujuba* ‘Huizao’ Wine.

The fermentation process of Xinjiang *Ziziphus jujuba* ‘Huizao’ wine was carried out as follows: *Ziziphus jujuba* ‘Huizao’ fruits → washing → steaming and crushing → solid–liquid ratio adjustment → sugar and acidity regulation → yeast activation → inoculation → static fermentation → distillation → finished ‘Huizao’ wine. The detailed technological flow is shown in Figure 1.

Briefly, *Z. jujuba* ‘Huizao’ fruits were washed, steamed, and crushed to obtain a semi-solid jujube paste. The paste was then mixed with purified water at a solid-to-liquid ratio of 1:2.5 (*w*/*v*, g:mL). After adjustment of sugar content and acidity, activated yeast cultures were inoculated into the prepared jujube substrate. The fermentation was carried out statically at 28 °C until the end of the fermentation period. After fermentation, the fermented mash was distilled to obtain the final ‘Huizao’ wine samples for subsequent physicochemical, volatile compound, and sensory analyses.

#### 2.2.2. Key Operating Points

Ingredient adjustment: The semi-solid *Ziziphus jujuba* ‘Huizao’ paste was mixed with purified water at a solid-to-liquid ratio of 1:2.5 (*w*/*v*), corresponding to 1 g of jujube paste mixed with 2.5 mL of purified water. Sucrose and food-grade citric acid were added to adjust the total sugar content to 180 g/L and total acidity (as citric acid) to 3.50 g/L, respectively.

Yeast activation: The yeast strains preserved at −80 °C were thawed at 4 °C for 20 min, and then 50 μL of the thawed culture was spread on YPD solid medium and incubated at 28 °C for 2–3 days. A single colony was picked and streaked twice on YPD solid medium for purification. The purified colony was inoculated into 50 mL YPD liquid medium and cultured at 28 °C with shaking at 180 rpm for 12 h to prepare seed culture; this liquid activation step was repeated twice [11].

Inoculation and fermentation: The activated yeast seed culture was inoculated into the prepared ‘Huizao’ juice at an inoculum size of 10% (*v*/*v*). Static fermentation was performed at 28 °C in a constant-temperature incubator. Fermentation was carried out in 250 mL sealed glass bottles containing 150 mL of prepared jujube juice, with a headspace ratio of approximately 1:1.5. All fermentation treatments were performed in triplicate biological replicates.

#### 2.2.3. Determination of Yeast Growth Characteristics and Stress Tolerance

Colony and cellular morphology: Yeast strains were streaked on YPD solid medium and incubated at 28 °C for 48 h; colony morphology (color, shape, surface smoothness, edge) was observed and recorded. The cellular ultrastructure was examined by scanning electron microscopy (SEM) according to the method of Wang et al. [12] with minor modifications.

Growth curve determination: Purified yeast colonies were inoculated into 10 mL YPD liquid medium and cultured at 28 °C with shaking at 180 rpm for 36 h. The optical density at 600 nm (OD_600_) was measured every 2 h using a UV-visible spectrophotometer, with sterile YPD liquid medium as the blank control. Growth curves were plotted with fermentation time as the abscissa and OD_600_ value as the ordinate.

Stress tolerance assay: Alcohol, acid, and sugar tolerance of yeast strains were determined using modified YPD liquid medium. For alcohol tolerance, ethanol concentrations were set at 6%, 8%, 10%, 12%, and 14% (*v*/*v*); for acid tolerance, citric acid concentrations were 10, 15, 20, 25, and 30 g/L; and for sugar tolerance, sucrose concentrations were 100, 150, 200, 250, and 300 g/L. Activated yeast culture (1 mL) was inoculated into each stress medium and incubated at 28 °C for 48 h. Gas production (bubble formation) in Duhamel tubes was used to evaluate tolerance: “−” = no bubbles, “+” = slight bubble formation, “++” = moderate bubble formation, “+++” = abundant bubble formation, “++++” = complete buoyancy of the Duhamel tube [13].

#### 2.2.4. Molecular Biological Identification of Yeast Strains

Yeast strains preserved at −80 °C were inoculated into YPD liquid medium and cultured at 28 °C with shaking at 180 rpm for 12–14 h. Genomic DNA was extracted, and the 26S rDNA gene was amplified by polymerase chain reaction (PCR). The PCR products were sequenced, and sequence alignment was performed using BLAST 2.16.0 on the NCBI database (https://www.ncbi.nlm.nih.gov/ accessed on 22 January 2026). A phylogenetic tree was constructed using MEGA 12 software with the neighbor-joining method, based on the method of Sun et al. [14].

### 2.3. Determination of Physicochemical Properties

The physicochemical properties of ‘Huizao’ wine samples (alcohol content, total sugar, total acidity) were determined according to the Chinese national standard GB/T 15038-2006 General Analytical Methods for Wine and Fruit Wine [15]. Soluble solids content was measured using a handheld refractometer (Atago, Tokyo, Japan) at 25 °C, and the results were expressed as °Brix. All determinations were performed in triplicate.

### 2.4. Volatile Compound Determination

#### 2.4.1. GC-FID

Gas chromatography (GC) was employed to determine the volatile flavor compounds in eight types of jujube wine. The content of each volatile compound was calculated using the internal standard method.

Equipment model: PANNA/A91 Gas chromatograph, EPU Chemical; Column: LZP-930 (50 m × 0.32 μm × 1.0 mm); Carrier gas: Nitrogen; Split ratio: 10:1; Carrier gas flow rate: 1 mL/min; Injection volume: 1 μL; Inlet temperature: 250 °C; Gas-Chromatography interface temperature: 250 °C; Temperature program: Initial temperature 50 °C, hold for 8 min, then ramp at 5 °C/min to 150 °C, hold for 15 min; Tailflow gas flow rate: 15 mL/min; Air flow rate: 400 mL/min; Hydrogen flow rate: 30 mL/min.

#### 2.4.2. HS-SPME-GC-MS Conditions and Methods

Headspace solid-phase microextraction (HS-SPME) was performed as follows: Briefly, 5 mL of jujube wine sample was transferred into a 20 mL headspace vial, and 150 μL of 2-octanol solution (25.44 mg/L in chromatographic grade ethanol) was added as an internal standard. The sample was pre-incubated in a water bath at 60 °C for 10 min. A DVB/CAR/PDMS fiber (50/30 μm, 1 cm length, Supelco, Bellefonte, PA, USA) was then inserted into the headspace of the vial for adsorption of volatile compounds for 60 min. After extraction, the fiber was immediately introduced into the GC inlet for thermal desorption at 250 °C for 5 min. The HS-SPME procedure was referenced and modified according to previous studies [16,17].

GC-MS analysis was carried out using a Shimadzu TQ8040 gas chromatograph coupled with a Shimadzu 2030 mass-selective detector (Shimadzu, Kyoto, Japan). The separation was performed on an HP-INNOWAX capillary column (60 m × 0.25 mm × 0.25 μm). The injection was performed in splitless mode with a solvent delay of 3 min. High-purity helium was used as the carrier gas at a constant flow rate of 1.0 mL/min. The temperatures of the injection port, ion source, and transfer line were set to 250 °C. The oven temperature program was as follows: initial temperature 40 °C (held for 1 min), increased to 100 °C at 3 °C/min (held for 1 min), and then raised to 230 °C at 5 °C/min. Electron impact ionization (EI) was performed at 70 eV, and the ion source temperature was maintained at 250 °C. Mass spectra were acquired in full-scan mode over the *m*/*z* range of 35–550. The GC-MS parameters were optimized based on the methods reported in the literature [16,17].

Volatile compounds were identified by comparing their mass spectra with those in the NIST 20 mass spectral library (National Institute of Standards and Technology, Gaithersburg, MD, USA). Compounds were considered tentatively identified when the match similarity index was greater than 80%. Volatile compounds were classified into esters, alcohols, acids, aldehydes/ketones, alkenes, and lactones according to their dominant functional groups and chemical structures. The relative contents of volatile compounds were calculated by semi-quantification using 2-octanol as the internal standard. Because authentic standards were not available for all volatile compounds, compounds identified only by NIST library matching were regarded as tentatively identified rather than fully confirmed.

### 2.5. Sensory Analysis

Sensory evaluation was conducted using a scoring system based on and modified from the national standard [15] (Table 1). A total of 30 trained panelists (15 males and 15 females, aged 20–50 years) participated in the sensory assessment of the distilled jujube wine samples. All panelists were specialized evaluators familiar with fruit wine sensory standards. Training included the recognition of jujube aroma, fruity, floral, and alcoholic notes, as well as calibration with standard wine samples. All samples were evaluated in triplicate biological replicates. Scores were averaged across panelists and replicates. The intraclass correlation coefficient (ICC) was used to verify the consistency and reliability of the panel, and the ICC value was greater than 0.85, indicating excellent evaluation consistency.

### 2.6. Statistical Analysis

Each indicator was determined from three biological replicates, and statistical analysis was performed using Excel 2019. The data were expressed as mean ± standard deviation. The graphical analysis of the data was conducted using Origin 2024 and the online platform Chiplot. Significance analysis was conducted using SPSS 17.0, and the significance level was set at *p* = 0.05.

## 3. Results

### 3.1. Morphological Traits and Biological Properties of Strains

#### 3.1.1. Growth Dynamics Curve, Tolerability Analysis and Biological Identification

**Figure 2:**
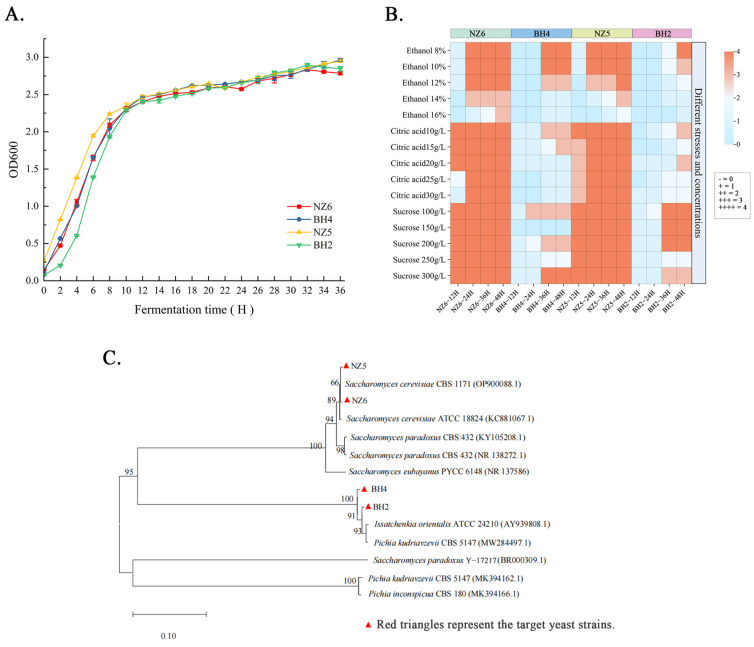
Growth Characteristics, Environmental Tolerance, and Phylogenetic Relationships of Diverse Yeast Strains. (**A**) Dynamic growth curve of yeast; (**B**) Heatmap analysis of tolerance among different yeast strains; (**C**) Phylogenetic tree of 4 yeast strains based on 26S rDNA gene sequences.

As shown in Figure 2A, all four yeast strains exhibited typical microbial growth trends in YPD medium. Among them, NZ5 had the shortest lag phase (<2 h) and the highest stationary-phase biomass (OD_600_ ≈ 2.60). NZ6 and BH4 followed similar growth patterns, where BH2 had a longer lag phase (4 h) but ultimately showed no significant difference in final biomass. Subsequent fermentation experiments will uniformly use late-log phase cultures of each strain as inoculum to ensure high yeast viability.

Tolerance is a core indicator of yeast strains’ adaptation to fermentation environments, directly determining their fermentation stability under stress conditions such as high sugar, high acidity, and high alcohol content. Tolerance profiles were visualized as a heat map (Figure 2B), which revealed NZ6 and NZ5 as highly tolerant strains with superior performance under citric acid, sucrose stress, and medium–low ethanol concentrations, making them suitable fermentation strains for high-stress environments typical of jujube fermentation. In contrast, BH4 showed markedly weaker tolerance, especially under high-ethanol and high-sugar conditions, consistent with its classification as a non-wine yeast. Most non-wine yeasts demonstrate weak growth capabilities in fermentation environments due to osmotic stress, high ethanol concentrations, temperature fluctuations, and SO_2_ exposure during fermentation [18].

Phylogenetic analysis based on 26S rDNA gene sequences (Figure 2C) revealed that NZ5 and NZ6 clustered with *Saccharomyces cerevisiae* type strains CBS 1171 and ATCC 18824, indicating close genetic affiliation and suggesting inheritance of typical *Saccharomyces cerevisiae* fermentation traits [19]. In the second clade, strains BH4 and BH2 formed a well-supported subclade with *Pichia kudriavzevii* CBS 5147 (synonym: *Issatchenkia orientalis* ATCC 24210). The high bootstrap support and close sequence similarity confirm that both BH4 and BH2 belong to the species *Pichia kudriavzevii*. This reflects the metabolic diversity characteristics of non-*Saccharomyces* yeasts and their potential contribution to unique flavor complexity [20].

The 26S rDNA sequences were deposited in the GenBank database under the following accession numbers: NZ5 (PZ386133), NZ6 (PZ386134), BH4 (PZ386131), and BH2 (PZ386132).

#### 3.1.2. Morphological Characteristics

**Figure 3:**
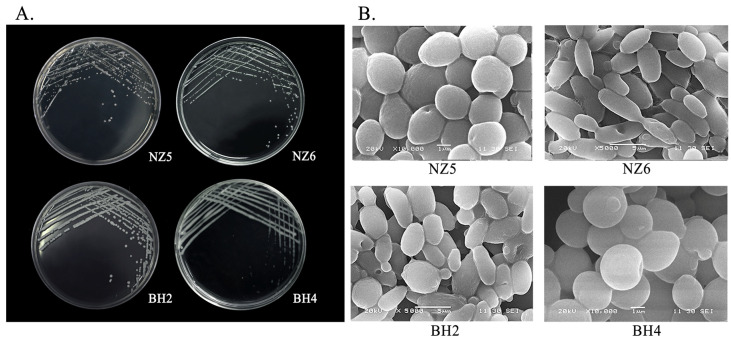
Morphological Observation of Different Yeast Strains. (**A**) Streak plate of yeast colonies; (**B**) Scanning electron micrograph (SEM) of yeast.

The morphological characteristics and cellular structure of a strain form the basis for its adaptation to the fermentation environment and the execution of its metabolic functions (Table 2 and Figure 3). NZ5, NZ6, and BH2 colonies appear white to pale yellow, circular, with smooth surfaces and well-defined edges. These morphological characteristics align with the *Saccharomyces cerevisiae* phenotype. Such morphology facilitates dispersion in liquid fermentation and enhance substrate accessibility [21]. Conversely, BH4 colonies are milky white, flat and elliptical, and slightly wrinkled surfaces and irregular edges, exhibiting a distinctive morphology. This distinctive morphology aligned with the traits of *Issatchenkia* species. Morphological variations in such non-*Saccharomyces* yeasts are often associated with the specificity of their metabolic pathways, laying the groundwork for the diversity of flavor compounds they synthesize [22]. Under electron microscopy, cells of all strains are predominantly round or oval, and all exhibit budding reproduction, indicating active metabolic states.

### 3.2. Fermentation Capability Analysis

**Figure 4:**
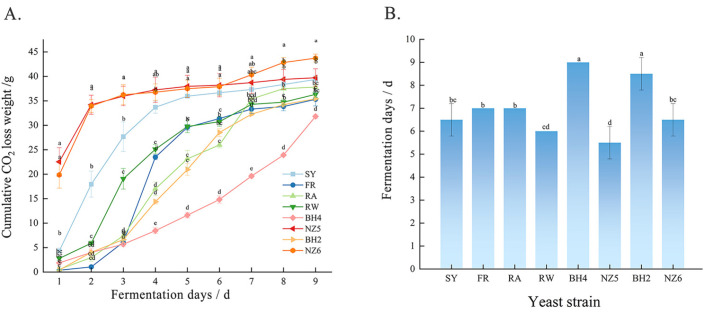
Fermentation capacity of different yeast strains. (**A**) Cumulative CO_2_ loss weight; (**B**) Fermentation days. Note: Different lowercase letters above the bars indicate statistically significant differences among different yeast strains (*p* < 0.05). Corresponding numerical values are provided in Supplementary Table S1.

Figure 4 summarizes the fermentation performance of all strains.

The fermentation performance of strains NZ5, NZ6, BH4, and BH2 significantly outperformed the reference commercial yeast. By the 9th day of fermentation, the cumulative CO_2_ loss of NZ5 and NZ6 exceeded 40 g, which was 14% higher than that of the commercial yeast FR, while BH4 and BH2 reached 35–40 g. Numerical data including the cumulative CO_2_ release corresponding to Figure 4A are listed in Supplementary Table S1. Fermentation duration varied among strains (Figure 4B). NZ5 and NZ6 required only 6 days, representing (3/4) three-quarters of the time required by commercial strain RW. BH4 and BH2 took 7 days, shortening SY’s duration by 12.5%. Among these, NZ5 and NZ6 demonstrated outstanding comprehensive fermentation performance advantages. This enhanced efficiency is attributed to rapid growth onset and high cell biomass, enabling accelerated glycolysis and CO_2_/ethanol production. Simultaneously, its robust stress tolerance maintains stable metabolic activity during fermentation, preventing arrest due to environmental stress [23].

### 3.3. Tracking and Monitoring of Basic Physical and Chemical Indicators During Fermentation

**Figure 5:**
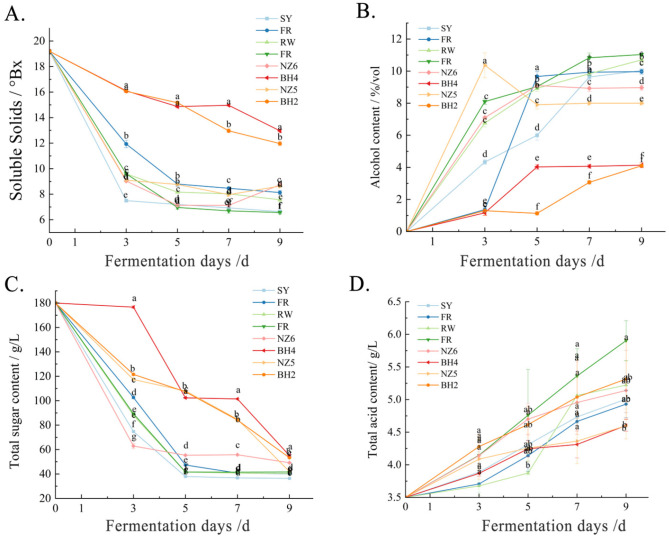
Physicochemical index monitoring of fermentation broths by different yeast strains. (**A**) Soluble solids; (**B**) Alcohol content; (**C**) Total sugar content; (**D**) Total acidity. Note: Different lowercase letters indicate statistically significant differences among different yeast strains at the same fermentation day (*p* < 0.05). The alcohol content in (**B**) refers to ethanol content (% *v*/*v*), excluding methanol and higher alcohols. Corresponding numerical values are provided in Supplementary Tables S2–S5.

Figure 5 illustrates the changes in soluble solids, alcohol content, total sugar and total acidity.

The rates of decline in soluble solids (Figure 5A) and total sugar content (Figure 5C) directly correlate with the strain’s sugar metabolism capacity. NZ6, SY, and FR exhibited the fastest sugar consumption rates, indicating higher activity of glycolysis pathway enzymes such as hexokinase and pyruvate kinase. In contrast, BH4 and BH2 exhibited slower sugar consumption rates, consistent with their weaker sugar tolerance. This slower metabolism contributed to their lower alcohol yields (Figure 5B). Although the FR strain achieved the highest final alcohol content, its extended fermentation duration and elevated total acidity reduced its overall process efficiency relative to NZ5 and NZ6.

Laboratory-preserved strains maintained significantly lower acidity levels, averaging 9.1% lower than commercial strains (Figure 5D), and their acidity remained consistently lower throughout the fermentation process. Since jujubes are naturally rich in organic acids, excessive acid formation during fermentation can intensify sourness and negatively influence flavor. Thus, the lower acid production by NZ5, NZ6, BH4, and BH2 contributes to a more harmonious sweet–sour balance [24].

Detailed numerical data for Figure 5, including soluble solids, alcohol content, total sugar, and total acidity at each fermentation time point, are available in Supplementary Tables S2–S5.

### 3.4. Key Volatile Substance Analysis (GC-FID)

**Figure 6:**
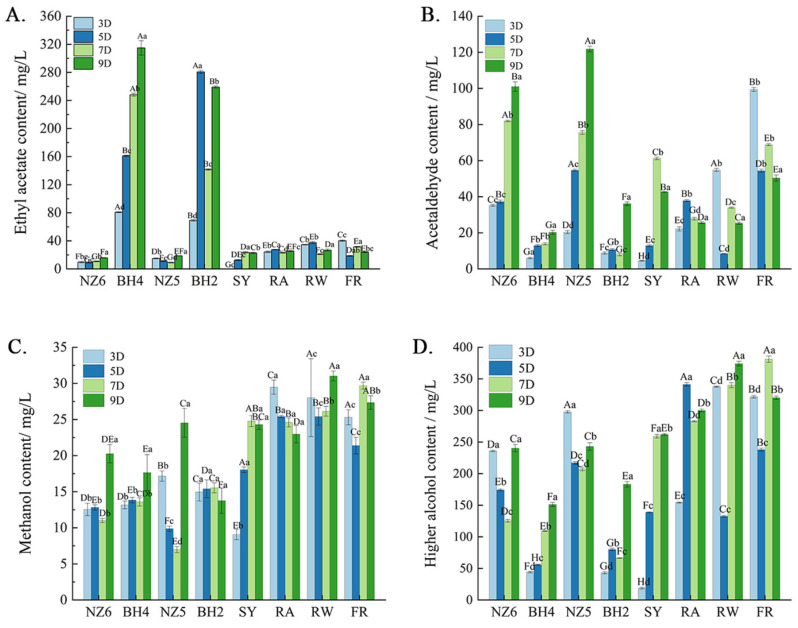
The concentration of key volatile compounds in jujube wines. (**A**) Ethyl acetate content; (**B**) Acetaldehyde content; (**C**) Methanol content; (**D**) Higher alcohol content. Note: Different uppercase letters indicate statistically significant differences among different yeast strains at the same fermentation day (*p* < 0.05); different lowercase letters indicate statistically significant differences at different fermentation days for the same yeast strain (*p* < 0.05). The total higher alcohols in this figure consist of n-propanol, isobutanol, isopentanol, n-butanol and 2-phenylethanol, excluding ethanol, methanol and acetaldehyde. Corresponding numerical values are provided in Supplementary Tables S6–S9.

Volatile compounds produced during yeast fermentation are important indicators of both sensory quality and safety in alcoholic beverages. In this study, ethyl acetate, acetaldehyde, methanol, and higher alcohols were selected as key volatile compounds to evaluate the aroma-forming capacity and safety-related characteristics of different yeast strains [25,26,27,28]. Figure 6 presents the key volatile compound analysis results for jujube wines fermented by different yeast strains.

As shown in Figure 6, significant strain-dependent differences were observed in the levels of key volatile compounds. Ethyl acetate increased during fermentation in nearly all samples. Among the tested strains, BH4 showed the strongest ethyl acetate-producing capacity, reaching 314.92 mg/L on day 9, which was more than 13-fold higher than that of commercial yeast FR (24.09 mg/L) and substantially higher than those of the other commercial control strains.

For acetaldehyde, NZ6 and NZ5 showed relatively higher levels, whereas BH2 and BH4 produced lower levels. Regarding methanol, NZ5 showed the lowest level on day 7, indicating a favorable safety-related profile at this fermentation stage; however, methanol formation should still be monitored during fermentation and scale-up production. For higher alcohols, RW showed the highest content on day 9, reaching 374 mg/L, whereas BH4 and BH2 produced lower levels of 109 mg/L and 66 mg/L, respectively, resulting in a softer wine style.

Collectively, BH4 stood out as a strong aroma-forming strain due to its high ethyl acetate production and relatively low levels of acetaldehyde and higher alcohols. In contrast, NZ5 showed the lowest methanol level on day 7; however, this advantage was not maintained throughout the later fermentation stage, and methanol formation should therefore be carefully monitored during fermentation optimization. Quantitative data corresponding to the volatile compounds shown in Figure 6 are provided in Supplementary Tables S6–S9.

### 3.5. HS-SPME-GC-MS

To further characterize the overall aroma profiles of wines fermented by different yeast strains, HS-SPME-GC-MS was used to analyze volatile flavor compounds in the eight wine samples. Detailed visualizations of compound classification, content distribution, heatmap clustering, common volatile compounds, and PCA analysis are provided in Supplementary Figures S1–S4.

A rich spectrum of volatile components was identified in the eight jujube wine samples via HS-SPME-GC-MS analysis (Figure S1A). The detected volatiles were mainly classified into esters, alcohols, aldehydes and ketones, organic acids, alkenes, and lactones. The indigenous yeast strains BH4 and BH2 presented higher volatile compound diversity than other tested strains and commercial yeast controls, and a certain number of volatile substances were shared by all fermentation groups (Figure S1B). The distribution and proportion of different compound categories are illustrated in Figures S1C and S2A, among which esters and alcohols dominated the overall volatile profiles. Esters occupied the predominant proportion in all samples (Figure S2B), with indigenous strains showing markedly higher ester accumulation than commercial yeasts, while alkenes and lactones maintained a low abundance level with slight differences across treatments.

Volatile compound heatmap clustering clearly divided all jujube wine samples into three distinct groups (Figure S3). The clustering results revealed obvious differences in volatile metabolic profiles among yeast strains, with the volatile characteristics of the preserved non-*Saccharomyces* strains BH4 and BH2 distinctly separated from *Saccharomyces cerevisiae* strains and commercial yeast controls.

As important aroma-active substances, esters are mainly generated by yeast metabolism and aging reactions, and endow wines with fruity and floral sensory attributes [29]. Ester composition and proportion presented significant strain-dependent differences in this study. Overall, preserved yeast strains possessed higher relative ester abundance than most commercial strains, and each strain had its own dominant ester components that could provide typical fruity and floral aroma notes for jujube wine [30].

The composition and accumulation of aroma-related alcohols also varied greatly among different fermentation groups (Figure S3). Preserved strains, especially BH4 and BH2, accumulated abundant floral characteristic alcohols, which were conducive to enhancing the floral aroma complexity of jujube wine. It should be noted that some volatile components were preliminarily identified based on mass spectral library matching, so the qualitative results should be interpreted cautiously.

Organic acids act as key precursors for ester synthesis and profoundly affect wine aroma complexity and taste balance [31]. BH2 and BH4 exhibited richer organic acid profiles and higher accumulation of characteristic fatty acids, while NZ5 and NZ6 produced unique organic acid components. By comparison, commercial yeast strains showed a simpler composition of organic acids.

The profiles of aldehydes and ketones also displayed remarkable strain-specific differences (Figure S3). Preserved yeast strains had higher species diversity and abundance of aldehydes and ketones than commercial controls, while commercial yeasts showed relatively limited composition and lower overall accumulation of such substances.

To further explore the shared volatile characteristics across the eight jujube wine samples, the common volatile components present in all treatments were analyzed (Figure S4A). Obvious differences in the accumulation of shared volatile components were observed between preserved indigenous strains and commercial yeast groups. PCA based on these common volatiles further differentiated the aroma compositional profiles of the eight yeast strains (Figure S4B). Commercial yeast strains were clearly separated from preserved strains, while BH4 and BH2 presented unique distribution features, confirming obvious strain-specific differences in volatile metabolic characteristics.

Overall, HS-SPME-GC-MS analysis demonstrated distinct aroma metabolic differences between preserved yeast strains and commercial controls. The preserved strains, particularly BH4 and BH2, exhibited higher volatile compound diversity and richer accumulation of aroma-related components, while commercial yeasts showed unique compositional characteristics in ester formation. These findings provide a theoretical reference for screening excellent yeast strains and optimizing fermentation technology to improve the flavor quality of jujube wine.

### 3.6. Sensory Analysis of the Eight Jujube Wines

**Figure 7:**
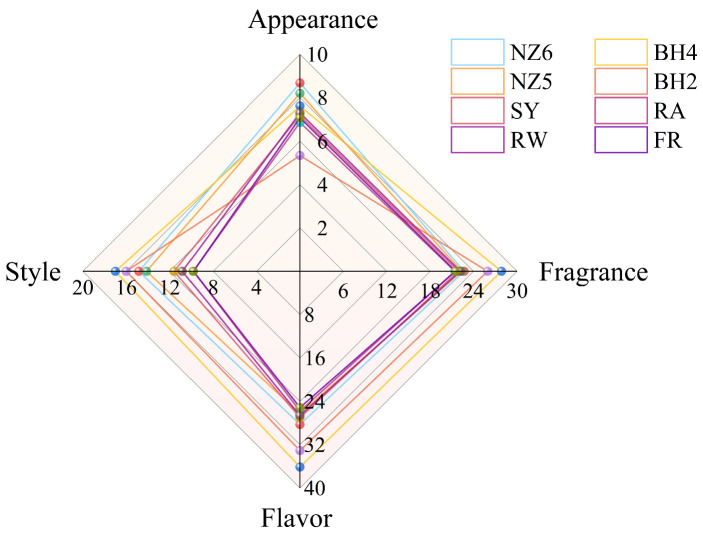
Sensory evaluation radar chart.

Sensory evaluation scores wines based on four aspects: appearance, fragrance, flavor, and style. Results are shown in Figure 7.

As shown in Figure 7, sensory evaluation showed significant differences in the sensory quality of jujube wine fermented by the eight yeast strains (*p* < 0.05). Among them, the non-*Saccharomyces* strains BH4 and BH2 achieved the best performance in aroma, flavor and overall style, ranking as the top two in comprehensive sensory scores. By contrast, NZ5 and NZ6 showed moderate sensory performance with acceptable appearance but insufficient aroma complexity. All commercial yeast strains presented relatively weak sensory performance across most indicators.

Overall, the four laboratory-preserved indigenous yeasts were markedly superior to commercial strains in sensory profiles of jujube wine fermentation, and better endowed the wine with richer aromatic complexity and typical flavor characteristics.

## 4. Discussion

This study systematically compared four laboratory-preserved yeast strains (NZ5, NZ6, BH4, and BH2) with four representative commercial strains used as controls (FR, RW, RA, and SY) during the fermentation of *Z. jujuba* ‘Huizao’ wine. The results showed that the preserved strains exhibited different advantages in growth behavior, fermentation kinetics, volatile compound formation, and sensory quality.

Specifically, NZ5 and NZ6 showed excellent growth characteristics, including a short lag phase (<2 h for NZ5), high stationary-phase biomass (OD_600_ ≈ 2.60 for NZ5), and strong tolerance to acidic, high-sugar, and moderate-alcohol environments. This rapid growth initiation may be attributed to efficient sugar transport systems and early activation of glycolytic enzymes, a phenomenon consistent with adaptive evolution observed in *S. cerevisiae* [32,33]. Their strong stress tolerance is consistent with previous findings that *Saccharomyces cerevisiae* can enhance osmotic stress resistance in high-sugar environments through multiple adaptive mechanisms, including the accumulation of osmoprotectants, membrane lipid remodeling, and the regulation of stress-related genes [34,35]. These traits enabled significantly enhanced fermentation efficiency; their fermentation cycles were shortened by 12.5–25% compared to commercial yeast strains, and cumulative CO_2_ release increased by 14%. This improved fermentation performance is comparable to that reported for selected *S. cerevisiae* strains in fruit wine fermentations [36,37].

In contrast, BH4 and BH2 displayed distinct advantages in volatile compound formation. BH4 showed the most complex volatile profile under the present analytical conditions. Its ethyl acetate level exceeded 300 mg/L, more than tenfold higher than those of the commercial control strains, and its phenethyl alcohol content reached 3.12 mg/L, 7.2 times that of commercial yeast RW. This level of ester production was higher than those reported for many non-Saccharomyces yeasts in fruit wine fermentations [38,39]. High ethyl acetate production is a characteristic feature of some non-*Saccharomyces* yeasts, including *Hanseniaspora*- and *Pichia*/*Issatchenkia*-related taxa [40,41], and the exceptional levels observed in BH4 suggest it may possess highly active acetyltransferases (ATF1/ATF2), the key enzymes responsible for ester synthesis [42,43]. The elevated phenethyl alcohol content is particularly noteworthy, as this compound imparts pleasant rose-like aromas and has been associated with the Ehrlich pathway utilizing L-phenylalanine as a precursor [44,45].

Moreover, the preserved strains produced additional aroma-related compounds. These terpenoid compounds are typically associated with secondary metabolism in yeasts and may be regulated by strain-specific terpene synthase genes [46,47]. Sensory evaluation further confirmed that the jujube wine fermented by preserved yeasts achieved significantly higher total scores than that by commercial yeasts. Among them, BH4 ranked first with a total score of 88.59, exhibiting excellent performance in aroma (27.84), flavor (36.13), and style (16.75). BH4 can be regarded as a promising aroma-enhancing candidate strain, whereas NZ5 and NZ6 are more suitable for efficient and stable fermentation. These sensory outcomes align well with the volatile compound analysis, reinforcing the well-established link between metabolic profiles and perceived quality in fermented beverages [48,49]. NZ5 and NZ6 showed clear advantages in fermentation efficiency, stress tolerance, and rapid fermentation initiation, but they produced relatively higher acetaldehyde levels. Although the acetaldehyde levels observed in this study did not indicate an obvious safety concern under the present experimental conditions, this compound should still be carefully considered because excessive acetaldehyde may negatively affect flavor quality by contributing pungent or grassy notes. Methanol is another important safety-related volatile compound in fruit wine fermentation. In this study, NZ5 showed relatively low methanol production, suggesting favorable safety performance, whereas methanol formation should still be monitored during future scale-up fermentation.

In contrast, BH4 and BH2 showed slower sugar consumption and lower ethanol-producing capacity, which are common characteristics of many non-*Saccharomyces* yeasts. However, they produced lower levels of acetaldehyde and total higher alcohols, contributing to a softer sensory profile.

From an industrial perspective, detailed information on the starter strains currently used in the Xinjiang *Ziziphus jujuba* ‘Huizao’ wine sector remains limited. Therefore, the commercial strains used in this study should be regarded as representative commercial control strains rather than confirmed dominant strains currently used in industrial ‘Huizao’ wine production. The preserved indigenous strains evaluated here should be considered promising candidate starters, and their industrial application still requires pilot-scale validation, process optimization, and long-term stability assessment. BH4 is a promising candidate for developing high-end, flavor-oriented jujube wines suitable for small-batch, high-value-added production. The use of non-Saccharomyces yeasts for enhancing wine aroma complexity has been increasingly investigated in fermented beverage production [50]. In contrast, NZ5 and NZ6, with their high fermentation efficiency and rapid initiation, may be more suitable for large-scale industrial fermentation aimed at shortening production cycles, consistent with industrial criteria for yeast selection [51]. Mixed or sequential fermentation combining aroma-producing non-*Saccharomyces* strains such as BH4 with highly fermentative *S. cerevisiae* strains such as NZ5 or NZ6 may provide a feasible strategy to improve both fermentation performance and aroma complexity. However, this approach remains to be experimentally verified in future studies. Overall, this work provides useful yeast resources and theoretical guidance for improving the quality, aroma diversity, and industrial development of Xinjiang *Ziziphus jujuba* ‘Huizao’ wine.

## 5. Conclusions

This study systematically evaluated four laboratory-preserved yeast strains (NZ5, NZ6, BH4, and BH2) and four commercial yeast strains for fermentation of Xinjiang *Ziziphus jujuba* ‘Huizao’ wine. The results demonstrated that the laboratory-preserved strains exhibited significant advantages over commercial strains in growth performance, stress tolerance, fermentation efficiency, volatile aroma formation, and sensory quality.

NZ5 and NZ6 showed excellent growth characteristics, including a short lag phase, high biomass, and strong tolerance to acidic, high-sugar, and moderate-alcohol environments. These advantages led to significantly improved fermentation efficiency, with fermentation cycles shortened by 12.5–25% and cumulative CO_2_ release increased by 14% compared with commercial strains.

In contrast, BH4 and BH2 exhibited outstanding advantages in aroma production. BH4 produced the richest and most diverse volatile compounds, including extremely high levels of ethyl acetate and phenethyl alcohol, which contributed to the best sensory performance.

Sensory evaluation confirmed that wines fermented by the laboratory-preserved yeasts achieved significantly higher overall scores than those fermented by commercial yeasts, among which BH4 ranked the highest.

In terms of safety-related compounds, NZ5 and NZ6 produced relatively higher acetaldehyde, but all values were within acceptable ranges. Meanwhile, NZ5 showed relatively low methanol production, indicating favorable safety characteristics. BH4 and BH2 produced lower acetaldehyde and higher alcohols, resulting in a softer and more harmonious flavor profile.

From an application perspective, BH4 is the most suitable strain for high-quality, aroma-oriented ‘Huizao’ wine production, while NZ5 and NZ6 are more appropriate for efficient, large-scale industrial fermentation. Mixed or sequential fermentation using BH4 combined with NZ5 or NZ6 represents a promising strategy to balance fermentation efficiency and aroma complexity.

Overall, this study provides high-quality yeast resources and theoretical support for improving the quality, flavor diversity, and industrial development of Xinjiang *Ziziphus jujuba* ‘Huizao’ wine.

## Figures and Tables

**Table 1:** Jujube wine sensory score standard.

Project	Scoring Criteria	Scoring
Appearance (10)	Crystal clear with a typical wine body color	9–10
	Transparent but with a subdued sheen	7–9
	Essentially transparent, with a faint haze.	5–7
	Noticeably cloudy or discolored	Under 5
Fragrance (30)	The pleasant aroma of flowers, fruits, dates, and wine	27–30
	Moderate jujube and wine aroma, but lacking richness.	24–27
	A faint aroma of dates and wine	21–24
	Odorless or even malodorous	Under 21
Flavor (40)	Delicate and mellow, with a lingering aftertaste	35–40
	Full-bodied texture with perfectly balanced sweetness and acidity	30–35
	Harsh and slightly pungent	25–30
	Flat or with a distinct off-flavor	Under 25
Style (20)	Distinctive style, vivid personality	17–20
	The style is relatively distinct but not sufficiently typical.	13–16
	Style is ambiguous or deviates	9–12
	No typical stylistic characteristics	Under 8

**Table 2:** Colony morphology of yeasts.

Number	Color	Shape	Smoothness	Edge
NZ5	White	Circular	Smooth	Smooth
BH4	Cream	Flat oval	Slightly wrinkled	Rough-edged
BH2	Pale yellow	Circular	Smooth	Smooth
NZ6	White	Circular	Smooth	Smooth

## Data Availability

The original contributions presented in the study are included in the article/Supplementary Materials. Further inquiries can be directed to the corresponding author.

## Supplementary Materials

The following supporting information can be downloaded at: https://www.mdpi.com/article/10.3390/microorganisms14061178/s1, Figure S1. HS-SPME-GC-MS Analysis of Eight Yeast Strains. Figure S2. Classification and content distribution of volatile flavor compounds. Figure S3. Heat map of volatile component content in different jujube wines. Figure S4. Analysis of Common Flavor Compounds in Different Yeasts. Table S1. Cumulative CO_2_ release weight of different yeast strains during fermentation. Table S2. Changes of soluble solids during fermentation of different yeast strains. Table S3. Changes of alcohol content during fermentation of different yeast strains. Table S4. Changes of total sugar content during fermentation of different yeast strains. Table S5. Changes of total acidity during fermentation of different yeast strains. Table S6. Concentration of ethyl acetate in jujube wine fermented by different yeast strains. Table S7. Concentration of acetaldehyde in jujube wine fermented by different yeast strains. Table S8. Concentration of methanol in jujube wine fermented by different yeast strains. Table S9. Concentration of higher alcohols in jujube wine fermented by different yeast strains.

## Abbreviations

The following abbreviations are used in this manuscript:

GC-FID	Gas Chromatography–Flame lonization Detector
HS-SPME-GC-MS	Headspace Solid-Phase Microextraction Gas Chromatography–Mass Spectrometry
PCA	Principal Component Analysis
