# Supplementary material for: Effects of Different Yeast Strains on Fermentation Characteristics, Volatile Flavor Compounds, and Sensory Quality of Xinjiang Ziziphus jujuba ‘Huizao’ Wine
Source: Microorganisms. 2026 May 23;14(6):1178. doi: 10.3390/microorganisms14061178 (PMC13302989; doi:10.3390/microorganisms14061178)
Supplement: Supplementary file 1 [file microorganisms-14-01178-s001.zip › Supplementary figures.pdf]

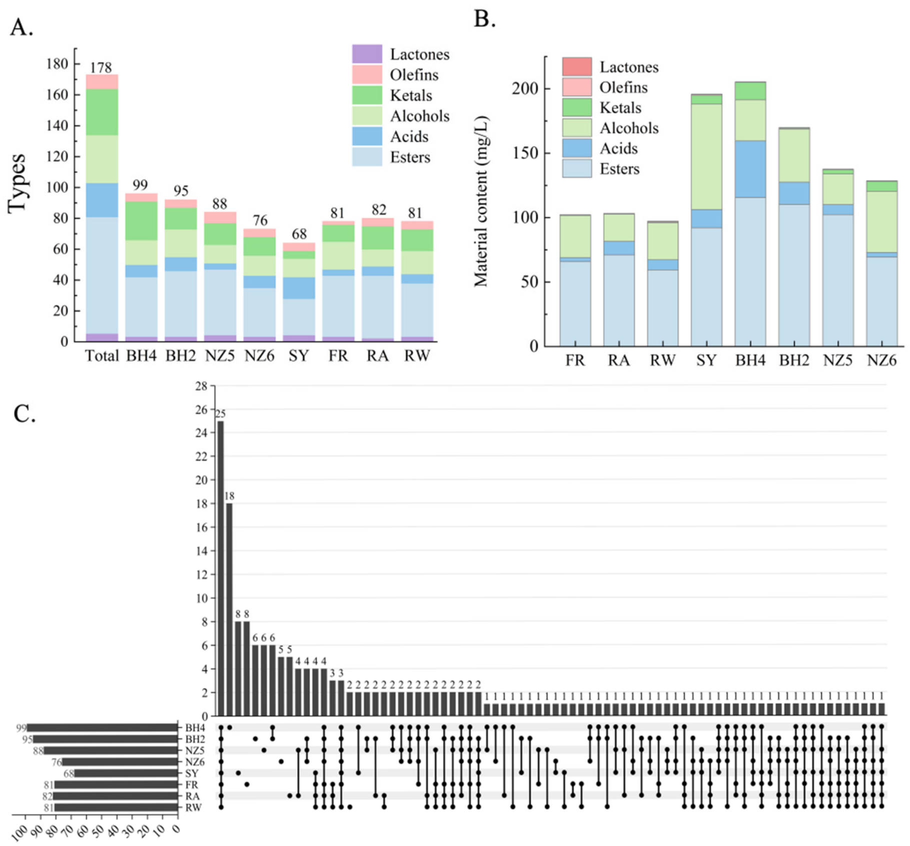

Figure S1. HS-SPME-GC-MS Analysis of Eight Yeast Strains. A.- Distribution of volatile flavor compound species among different yeast strains. B.-Total content of volatile flavor compounds in different yeast strains. C.-Upset plot of volatile flavor compounds for different yeast strains.

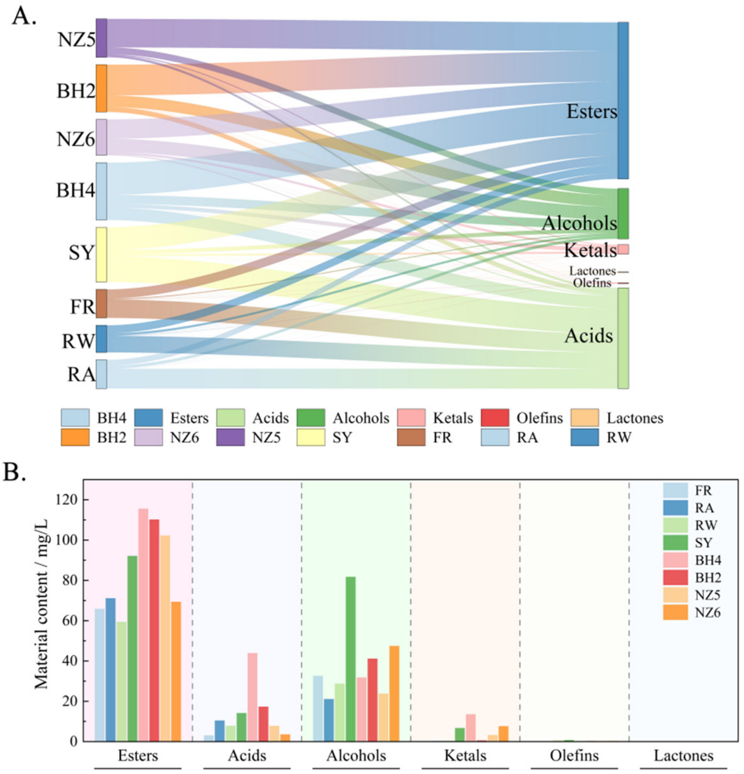

Figure S2. Classification and content distribution of volatile flavor compounds. A.-Sankey diagram correlating the number of flavor compounds across six categories with different wine samples. B.-Comparison chart of flavor compound classification and content.

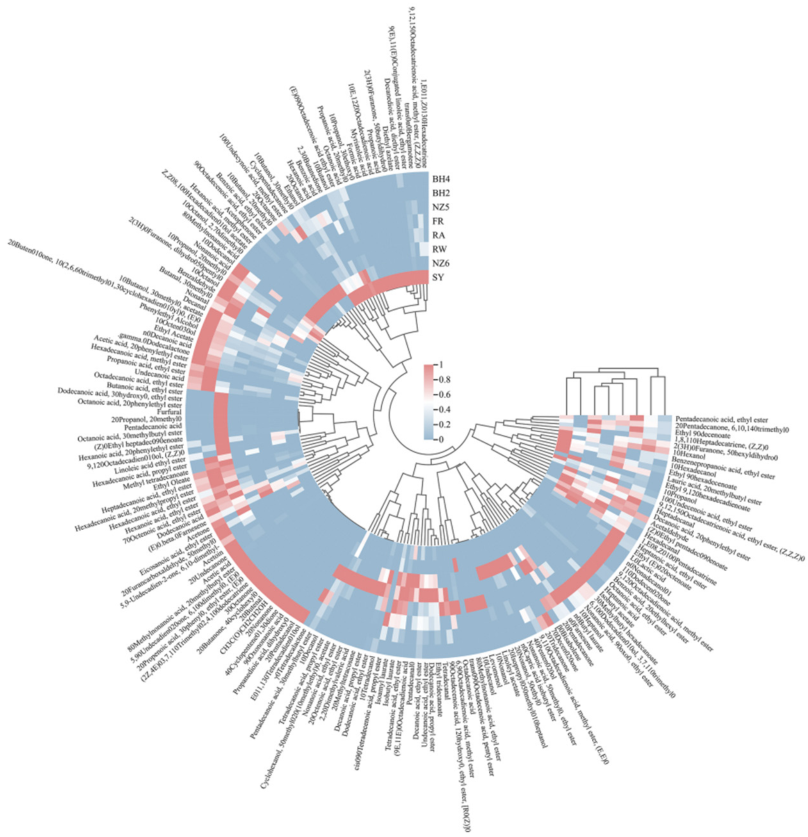

Figure S3. Heat map of volatile component content in different jujube wines.

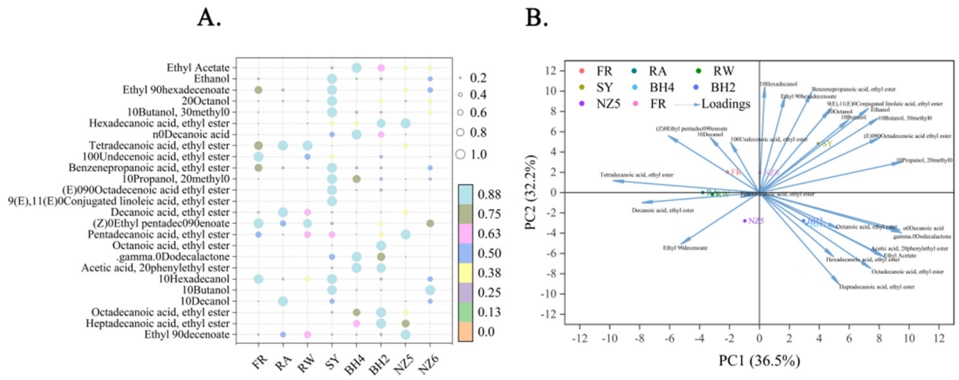

Figure S4. Analysis of Common Flavor Compounds in Different Yeasts. A.- Bubble chart of 22 common volatile components; B.- Principal Component Analysis (PCA) plot of common volatile substances.
