# Supplementary material for: Effects of Different Yeast Strains on Fermentation Characteristics, Volatile Flavor Compounds, and Sensory Quality of Xinjiang Ziziphus jujuba ‘Huizao’ Wine
Source: Microorganisms. 2026 May 23;14(6):1178. doi: 10.3390/microorganisms14061178 (PMC13302989; doi:10.3390/microorganisms14061178)
Supplement: Supplementary file 1 [file microorganisms-14-01178-s001.zip › Supplementary tables.pdf]

Table S1. Cumulative CO_2 release weight of different yeast strains during fermentation.

Days	SY	FR	RA	RW	BH4	NZ5	BH2	NZ6
2	17.96±2.67b	1.05±0.21d	2.96±0.75cd	5.90±0.47c	4.01±0.88cd	34.21±1.95a	4.03±0.20cd	33.94±1.31a
3	27.67±3.03b	6.10±0.16d	7.40±0.92d	19.06±2.11c	5.68±0.44d	35.97±1.96a	6.64±0.70d	36.25±2.03a
4	33.70±1.23b	23.49±0.52c	17.01±1.41d	25.11±0.96c	8.46±0.60e	37.25±2.56a	14.37±0.45d	36.77±1.68ab
5	35.97±0.26a	29.60±0.59b	23.14±1.73c	29.76±1.26b	11.62±0.54d	37.97±2.25a	20.95±1.22c	37.48±1.78a
6	36.67±0.60a	31.38±0.10b	25.99±0.60c	30.74±0.96b	14.83±0.74d	38.20±2.38a	28.53±0.81bc	37.90±1.72a
7	37.32±0.17abc	33.32±0.91d	35.40±2.66bcd	34.29±0.44cd	19.63±0.41e	38.73±2.21ab	32.29±0.30d	40.34±1.51a
8	38.35±0.66b	33.85±0.88c	37.50±2.00b	34.73±0.54c	23.92±0.13d	39.39±2.02b	34.23±0.51c	42.84±1.01a
9	39.38±0.88b	35.31±1.34c	37.82±2.19bc	36.33±0.60c	31.81±0.39d	39.73±1.83b	35.43±0.14c	43.74±0.81a

Note: The unit of cumulative CO_2 loss weight is g. Values in the same row followed by different lowercase letters indicate significant differences ($p < 0.05$).

Table S2. Changes of soluble solids during fermentation of different yeast strains.

Yeasts/Day	Original	3D	5D	7D	9D
SY	19.20	7.50±0.10e	7.20±0.20d	6.93±0.06ef	6.63±0.15f
RA	19.20	11.93±0.29b	8.80±0.17b	8.47±0.06c	8.13±0.15d
RW	19.20	9.60±0.20c	8.17±0.06c	8.03±0.06d	7.57±0.15e
FR	19.20	9.60±0.20c	6.97±0.15d	6.70±0.17f	6.57±0.12f
NZ6	19.20	9.03±0.06d	7.13±0.12d	7.13±0.12e	8.73±0.12c
BH4	19.20	16.10±0.10a	14.87±0.12a	14.97±0.06a	12.97±0.15a
NZ5	19.20	9.10±0.10d	8.77±0.06b	7.97±0.15d	8.63±0.12c
BH2	19.20	16.07±0.12a	15.17±0.12a	12.97±0.15b	11.97±0.15b

Note: The unit of soluble solids is °Bx. Values in the same column followed by different lowercase letters indicate significant differences ($p < 0.05$).

Table S3. Changes of alcohol content during fermentation of different yeast strains.

Yeasts/Day	Original	3D	5D	7D	9D
SY	19.20	7.50±0.10e	7.20±0.20d	6.93±0.06ef	6.63±0.15f
RA	19.20	11.93±0.29b	8.80±0.17b	8.47±0.06c	8.13±0.15d
RW	19.20	9.60±0.20c	8.17±0.06c	8.03±0.06d	7.57±0.15e
FR	19.20	9.60±0.20c	6.97±0.15d	6.70±0.17f	6.57±0.12f
NZ6	19.20	9.03±0.06d	7.13±0.12d	7.13±0.12e	8.73±0.12c
BH4	19.20	16.10±0.10a	14.87±0.12a	14.97±0.06a	12.97±0.15a
NZ5	19.20	9.10±0.10d	8.77±0.06b	7.97±0.15d	8.63±0.12c
BH2	19.20	16.07±0.12a	15.17±0.12a	12.97±0.15b	11.97±0.15b

Note: The unit of alcohol content is % vol. Values in the same column followed by different lowercase letters indicate significant differences ($p < 0.05$).

Table S4. Changes of total sugar content during fermentation of different yeast strains.

Yeasts/Day	Original	3D	5D	7D	9D
SY	180	74.84±0.27f	37.95±0.51g	36.84±0.37e	36.41±0.29f
RA	180	102.66±1.06d	47.39±0.53e	40.72±0.24d	40.28±0.17e
RW	180	87.95±0.44e	41.58±0.49f	40.72±0.24d	40.49±0.38e
FR	180	89.38±0.56e	41.58±0.46f	41.49±0.32d	41.63±0.35d
NZ6	180	62.89±2.22g	55.34±0.54d	55.74±0.19c	49.25±0.43c
BH4	180	176.62±1.08a	102.33±0.45c	101.48±0.18a	55.06±0.83a
NZ5	180	117.36±0.17c	108.02±0.14a	84.88±0.66b	42.05±0.50d
BH2	180	121.54±0.99b	107.35±0.27b	84.15±0.46b	53.35±0.17b

Note: The unit of total sugar content is g/L. Values in the same column followed by different lowercase letters indicate significant differences ($p < 0.05$).

Table S5. Changes of total acidity during fermentation of different yeast strains.

Yeasts/Day	Original	3D	5D	7D	9D
SY	3.5	3.89±0.31a	4.31±0.03ab	4.73±0.10a	5.01±0.21ab
RA	3.5	3.71±0.20a	4.14±0.24ab	4.67±0.07a	4.93±0.21ab
RW	3.5	3.68±0.15a	3.87±0.03b	5.06±0.28a	5.22±0.37ab
FR	3.5	4.15±0.23a	4.76±0.70a	5.36±0.42a	5.90±0.31a
NZ6	3.5	4.14±0.20a	4.70±0.24ab	4.96±0.62a	5.14±0.62ab
BH4	3.5	3.87±0.04a	4.25±0.12ab	4.31±0.20a	4.60±0.09b
NZ5	3.5	4.09±0.31a	4.26±0.22ab	4.36±0.35a	4.6±0.20b
BH2	3.5	4.28±0.18a	4.62±0.28ab	5.04±0.57a	5.32±0.61ab

Note: The unit of total acidity content is g/L. Values in the same column followed by different lowercase letters indicate significant differences ($p < 0.05$).

Table S6. Concentration of ethyl acetate in jujube wine fermented by different yeast strains.

Yeasts/Day	3D	5D	7D	9D
NZ6	9.77 ±1.09Fbc	8.98 ±0.63Gc	10.81 ±0.69Gb	15.85 ±0.46Aa
BH4	80.82 ±0.68Ad	161.29 ±0.94Bc	248.15 ±2.18Ab	314.92 ±10.31Bb
NZ5	15.01 ±0.68Db	11.30 ±0.87Fc	9.07 ±0.65Gd	18.72 ±0.21EFa
BH2	69.25 ±1.12Bd	280.68 ±2.35Aa	141.68 ±1.18Bc	258.99 ±1.52Bb
SY	0.00 ±0.00Gd	12.68 ±0.71DEc	23.64 ±1.01Da	22.95 ±0.78Cb
RA	24.33 ±1.19Eb	27.61 ±0.38Ca	23.06 ±0.51Cd	25.60 ±0.83EFc
RW	34.57 ±0.59Cb	37.57 ±1.54Eb	21.07 ±0.83Fc	26.76 ±1.51Da
FR	40.28 ±0.95Cc	18.61 ±0.65Da	31.55 ±0.57Ea	24.09 ±0.74Ebc

Note: The unit of ethyl acetate is mg/L. Uppercase letters indicate significant differences among different yeast strains at the same fermentation time point, while lowercase letters indicate significant differences for the same yeast strain at different fermentation time points ($p < 0.05$).

Table S7. Concentration of acetaldehyde in jujube wine fermented by different yeast strains.

Yeasts/Day	3D	5D	7D	9D
NZ6	35.24 ±0.48Cc	37.28 ±0.87Bc	81.99 ±0.39Ab	101.00 ±2.63Ba
BH4	6.06 ±0.44Ga	12.99 ±0.51Fb	13.92 ±0.57Fb	20.29 ±1.09Ga
NZ5	20.46 ±0.90Dd	54.69 ±0.58Ac	75.56 ±1.04Bb	121.80 ±1.53Aa
BH2	8.84 ±0.63Fc	10.75 ±0.50Gb	7.96 ±0.70Gc	36.16 ±1.00Fa
SY	4.57 ±0.19Hd	12.91 ±0.50Ec	61.22 ±0.69Cb	42.67 ±0.18Ba
RA	22.20 ±1.17Ec	37.86 ±0.57Bb	27.87 ±0.77Gd	25.68 ±0.59Da
RW	54.84 ±0.85Ab	8.41 ±0.15Cd	33.92 ±0.36Dc	25.35 ±0.55Ca
FR	99.41 ±1.12Bb	54.41 ±0.89Db	68.82 ±0.50Eb	50.39 ±1.68Ea

Note: The unit of acetaldehyde is mg/L. Uppercase letters indicate significant differences among different yeast strains at the same fermentation time point, while lowercase letters indicate significant differences for the same yeast strain at different fermentation time points ($p < 0.05$).

Table S8. Concentration of methanol in jujube wine fermented by different yeast strains.

Yeasts/Day	3D	5D	7D	9D
NZ6	12.55 ±0.86Db	12.82 ±0.37Eb	11.00 ±0.29Db	20.27 ±1.27DEa
BH4	13.17 ±0.54Db	13.84 ±0.37Eb	13.59 ±0.57CDb	17.65 ±2.48Ea
NZ5	17.17 ±0.70Bb	9.87 ±0.35Fc	6.99 ±0.42Ed	24.54 ±1.99Ca
BH2	14.96 ±1.24Ca	15.40 ±1.23Da	15.54 ±0.71Ca	13.76 ±1.75Ea
SY	9.08 ±0.71Eb	18.04 ±0.39Aa	24.77 ±0.77ABa	24.30 ±0.66BCa
RA	29.48 ±0.98Aa	25.41 ±0.15Bb	24.60 ±0.63Bc	22.96 ±1.21Dd
RW	28.04 ±5.40Ac	25.41 ±1.20Bc	26.14 ±0.68Bb	31.02 ±0.69Aa
FR	25.31 ±1.05Ac	21.39 ±1.14Cc	29.69 ±0.48Aa	27.34 ±0.94ABb

Note: The unit of methanol is mg/L. Uppercase letters indicate significant differences among different yeast strains at the same fermentation time point, while lowercase letters indicate significant differences for the same yeast strain at different fermentation time points ($p < 0.05$).

Table S9. Concentration of higher alcohols in jujube wine fermented by different yeast strains.

Yeasts/Day	3D	5D	7D	9D
NZ6	235.99 ±1.27Da	174.33 ±1.42Eb	125.38 ±2.11Dc	240.38 ±5.78Ca
BH4	44.21 ±1.15Fd	55.99 ±0.85Hc	109.23 ±0.42Eb	151.24 ±3.15Fa
NZ5	297.81 ±1.84Aa	217.08 ±2.13Dc	206.74 ±2.29Cd	243.07 ±5.96Cb
BH2	43.17 ±1.77Gd	80.11 ±1.83Gb	66.44 ±0.40Fc	183.09 ±4.34Ea
SY	18.88 ±1.31Hd	138.92 ±0.79Fc	259.16 ±2.79Fa	262.11 ±1.35Eb
RA	154.28 ±0.79Ec	341.24 ±2.75Aa	283.01 ±1.18Dd	299.98 ±2.22Db
RW	337.75 ±0.88Cd	132.56 ±1.29Cc	340.00 ±3.96Bb	374.14 ±3.70Aa
FR	321.81 ±2.13Bd	237.89 ±2.35Bc	381.24 ±4.95Aa	320.18 ±2.63Bb

Note: The unit of higher alcohols is mg/L. Uppercase letters indicate significant differences among different yeast strains at the same fermentation time point, while lowercase letters indicate significant differences for the same yeast strain at different fermentation time points ($p < 0.05$).
